# Heparin, Heparan Sulphate and Sepsis: Potential New Options for Treatment

**DOI:** 10.3390/ph16020271

**Published:** 2023-02-10

**Authors:** John Hogwood, Elaine Gray, Barbara Mulloy

**Affiliations:** 1 National Institute for Biological Standards and Control, Blanche Lane, South Mimms EN6 3QG, UK; 2 Sackler Institute of Pulmonary Pharmacology, Institute of Pharmaceutical Science, King’s College London, Stamford St., London SE1 9NH, UK

**Keywords:** heparin, heparin mimetics, heparan sulphate, sepsis, neutrophils, heparanase

## Abstract

Sepsis is a life-threatening hyperreaction to infection in which excessive inflammatory and immune responses cause damage to host tissues and organs. The glycosaminoglycan heparan sulphate (HS) is a major component of the cell surface glycocalyx. Cell surface HS modulates several of the mechanisms involved in sepsis such as pathogen interactions with the host cell and neutrophil recruitment and is a target for the pro-inflammatory enzyme heparanase. Heparin, a close structural relative of HS, is used in medicine as a powerful anticoagulant and antithrombotic. Many studies have shown that heparin can influence the course of sepsis-related processes as a result of its structural similarity to HS, including its strong negative charge. The anticoagulant activity of heparin, however, limits its potential in treatment of inflammatory conditions by introducing the risk of bleeding and other adverse side-effects. As the anticoagulant potency of heparin is largely determined by a single well-defined structural feature, it has been possible to develop heparin derivatives and mimetic compounds with reduced anticoagulant activity. Such heparin mimetics may have potential for use as therapeutic agents in the context of sepsis.

## 1. Introduction

Sepsis is described as a multi-organ failure syndrome caused by deregulated host defence against infection: see Sepsis (who.int) URL (accessed on 24 November 2022). In a 2017 global burden of diseases, injuries and risk factor study, 49 million people were estimated to contract sepsis which led to 11 million deaths globally every year [1]. Sepsis can be treated successfully with anti-microbials especially with early diagnosis and treatment. However, definitive diagnosis of sepsis is difficult. The updated international consensus on the definition of sepsis in 2016 [2] and the availability of guidance on diagnosis and treatment of sepsis associated pathological diseases such as disseminated intravascular coagulation (DIC) [3,4,5,6], improved the global harmonisation of clinical diagnosis, but sepsis related mortality and morbidity remains a top public health concern.

It is generally accepted that deranged inflammation and coagulation are the two major contributing factors to sepsis related pathology. Jarczak et al. [7] detailed the engagement of the complement and coagulation pathways in the manifestation of sepsis and the therapeutic options but concluded that the success of therapeutics such as steroid and immunoglobulins have not been conclusive and remained controversial. Microbes and associated endotoxins activate a number of cell types including endothelial cells and monocytes to generate procoagulant tissue factor. Concurrent stimulation of the proinflammatory complement pathways also triggers the contact phase of the coagulation cascade that further substantiates consumption of coagulation factors/inhibitors leading to thrombin generation and DIC if uncontrolled [8].

Investigations have also shown that binding of heparin to SARS-CoV-2 spike proteins may inhibit virus infectivity [9,10]. Indeed, clinical trials with nebulised heparin exploring the anti-inflammatory and anti-viral properties of heparin are ongoing [11,12,13,14]. Recent advances in heparin research indicated that heparin not only interacts with the coagulation cascades, but also with blood cells and numerous components of the complement system thereby influencing the innate immune responses to infection [15].

The subject of sepsis is addressed in several recent reviews of the non-anticoagulant properties of heparin [16,17], and the timely topic of possible roles for heparin mimetics in the treatment of SARS-CoV-2 has attracted attention [18,19]. The role of heparin in modulating the interplay of coagulation and inflammation in sepsis termed ‘thrombo-inflammation’ has also been explored very recently [8]. This current review aims to provide a short guide to the non-anticoagulant roles of heparin and its mimetics which may impact on the manifestation of sepsis and sepsis associated pathology by disrupting pathways dependent on cell surface and matrix heparan sulphate (HS).

**Figure 1:**
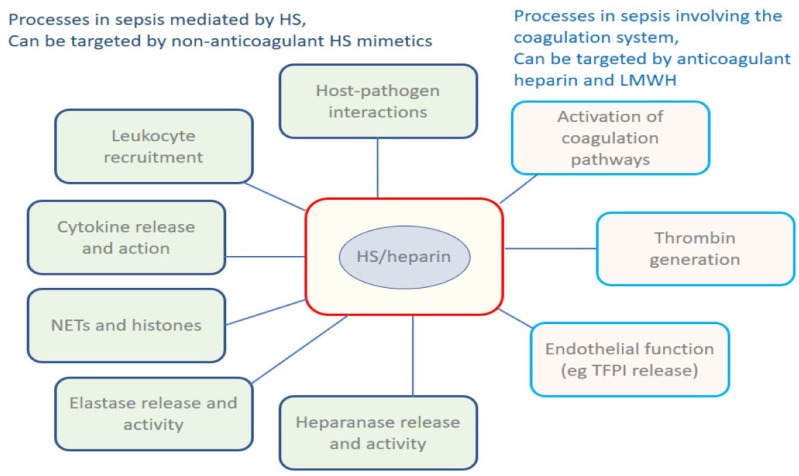
The aspects of sepsis that may be modified by the administration of heparin-based therapeutics can be divided into two categories, those for which the anticoagulant/antithrombotic properties of heparin are critical on the one hand, and those that are not dependent on anticoagulant action, but are based on disrupting HS-dependent processes. These processes are not independent of each other, and perturbation of one process will be reflected in all the others. Heparin and its relatives are well placed for development of treatment options in which anticoagulant and anti-inflammatory properties are combined to control the combination of excessive inflammatory and coagulation responses to infection that give rise to sepsis. Abbreviations; HS: heparan sulphate; LMWH: low molecular weight heparin; NETs: neutrophil extracellular traps; TFPI: tissue factor pathway inhibitor.

HS dependent processes in sepsis are summarised in Figure 1, which acts as a ‘graphical table of contents’. None of the processes shown are independent of one another; they interact in a complex web, disregarding the distinctions drawn by scientists between inflammation, immunity and coagulation.

## 2. Anticoagulant Action of Heparin in Sepsis

Invariably sepsis results in changes to the haemostasis system which requires clinical interventions [20] with a portion of patients presenting with DIC [21]. A substantial proportion (up to >50%) of all sepsis patients will develop thrombocytopenia [22,23], with platelet count correlating with severity [24]. Reduction in platelet levels is in part via reduced production [25] but also consumption/activation. During the early stages of sepsis, generation of pro-inflammatory markers leads to platelet activation and thrombin production [26]. Furthermore, platelet–neutrophil interaction, in the context of thrombo-inflammation [27], also consumes platelets and is described below. Heparin, and related mimetics, are antithrombotic agents [28,29] and could therefore attenuate thrombus formation through inhibition of activated coagulation factors [30]. With thrombin known to have targets outside coagulation, such as the complement pathway [31], attenuation would be beneficial. Release of cell surface HS anchored tissue factor pathway inhibitor (TFPI) [32] may have benefit in early or pre-sepsis to reduce coagulation, although the potential benefit of heparin in the later stages of sepsis may be limited due to sepsis disruption of this pathway [33]. The timing, therefore, of heparin or mimetics in sepsis treatment or prophylaxis may be critical for its antithrombotic activity.

Due to the procoagulant and prothrombotic nature of the early stages of sepsis, heparin and low molecular weight heparins have been recommended [34] and see https://b-s-h.org.uk/guidelines/guidelines/diagnosis-and-management-of-disseminated-intravascular-coagulation-1 URL (accessed on 24 November 2022) for prophylaxis and treatment of sepsis associated thrombosis and DIC. In this context heparin is being used as an anticoagulant/antithrombotic, dampening the consumption of plasmatic coagulation factors (and inhibitors). Heparin is recommended as an antithrombotic for the prevention of thrombosis in COVID 19 patients, with a number of clinical trials addressing the safety and efficacy of its use [34,35,36,37,38,39,40,41,42,43,44]. Recent meta-analyses on clinical trials of heparin and low molecular weight heparin (LMWH) in adult septic patients indicate both types of heparins reduced 28 day mortality and multiple organ failure events [45,46]. Furthermore, in the LMWH trials, reduction in levels of inflammatory markers such as tumour necrosis factor-α, and IL-6 was also observed [46]. Other heparinoids and mimetics such as danaparoid and pentosan polysulphate are also considered as treatment options to counteract not only the prothrombotic nature of infection, but also for their potential anti-viral and anti-inflammatory properties. Danaparoid, a mixture of heparan, dermatan and chondroitin sulphate, is a licensed antithrombotic, with indication for heparin induced thrombocytopenia. It has been shown in sepsis experimental models that danaparoid can inhibit systemic inflammation and prevent multiple organ failure [47,48], and it was proven to be superior to heparin when used in combination with antithrombin [49]. Danaparoid has also been used successfully in reported cases of vaccine induced immune thrombocytopenia and it is possible that the reduction in the proinflammatory C-reactive protein may have contributed to its success [50]. Pentosan polysulphate is a semi-synthetic sulphated xylan, once used as an antithrombotic and antilipemic agent and now licensed for treatment of interstitial cystitis in human and osteoarthritis in animals. Bertini et al. [51], have demonstrated that pentosan polysulphate can inhibit SARS-CoV-2 entry into vero cells indicating its potential as an anti-viral agent.

## 3. Non-Anticoagulant Action of Heparin and Sepsis

Heparan sulphate (HS), a member of the glycosaminoglycan family of linear sulphated polysaccharides, occurs as a major component of the glycocalyx, a dense carbohydrate-rich layer on the surface of cells. Heparin is a particularly highly sulphated type of HS found in the granules of mast cells. The structural feature in heparin that confers potent anticoagulant activity is a specific pentasaccharide sequence with high affinity for the serine protease inhibitor antithrombin; this motif is rare in HS. Details of the structure of heparin and HS and its relationship to biological properties have been reviewed [15,52,53].

### 3.1. Pathogen–Host Interactions

Cell-surface HS is used by many microbial pathogens, whether bacteria, viruses or parasites, as a host attachment factor [16,54]. This includes both Gram +ve and Gram −ve bacteria that are the most common infections leading to sepsis. Adherence of microbial pathogens to the host cells and matrix is an important aspect of virulence, as it mediates colonisation and infection [55]. In addition, adherence to host phagocytic cell surface HS can be a precursor to the phagocytosis of bacteria [56]. Treatment with exogenous heparin or its mimetics may be proposed based on a decoy mechanism, reducing direct microbe interaction with cell surface HS by competition.

#### 3.1.1. Bacterial Sepsis

Two of the most common causative organisms of bacterial sepsis are *Pseudomonas aeruginosa* (Gram −ve) and *Staphylococcus aureus* (Gram +ve). Both of these species can interact with several cell surface attachment receptors, including HS.

*Pseudomonas aeruginosa*: Long-term chronic *P. aeruginosa* lung colonization has been found to lead to a change in the structure of lung HS. Synthesized heparin-like HS competitors with low or absent anticoagulant activity reduced neutrophil recruitment and cytokine production in acute and chronic *P. aeruginosa* lung infection in mice [57], besides reducing bacterial burden.

The glycocalyx of at least some cell types is not uniform over the whole surface, leading to differences in, for example, the apical and basolateral surfaces of epithelial cells. Whereas *P. aeruginosa* utilises interactions between its pili and host *N*-glycans to attach to and invade the apical surface of fully polarised epithelial cells, at the basolateral surface interactions between bacterial flagella and host HS perform the same function [58,59].

*P. aeruginosa* expresses a heparinase that is essential for virulence; when the enzyme is mutated, mortality in thermally injured and infected mice is drastically reduced, and the bacterium is unable to spread systemically to the liver and spleen [60].

*Staphylococcus aureus*: *S. aureus* is known to bind to heparin; it can adhere to heparin-coated medical devices. The virulence factor protein A of *S. aureus* NCTC8325 is a heparin binding protein, and its mutation or deletion reduces the heparin-binding capacity of the bacterium [61]. The major autolysin of *S. aureus* has also been shown to mediate interaction with heparin, as well as host cellular component proteins such as fibronectin and thrombospondin [62]. *S. aureus* can bind to and be internalized by enterocytes in the intestine in a manner independent of its best-described attachment mode (through fibronectin and integrins). This can be inhibited by heparin, and exogenous HS, strongly implying that HSPGs act as receptor or co-receptor [63].

There has been a case report of the effective removal of *S. aureus* from the blood of an infected haemodialysis patient by the use of an extracorporeal blood filter incorporating heparin immobilised onto polyethylene beads, followed more recently by a small trial [64,65]. The effective use of heparin as a direct competitor with HS in anti-bacterial therapy requires delivery of heparin in high concentration to the location of the infection, and in this case extracorporeal circulation delivered the infection to the therapy. Similar treatment has been investigated also for reduction in viral load in HSV-2 associated liver failure [66], and for *Streptococcus pneumoniae* pneumonia in a baboon model [67].

#### 3.1.2. Viral Sepsis

A review published in 2018 regarded a diagnosis of viral sepsis as being very rare [68], though a substantial minority of sepsis cases were culture negative, suggesting a non-bacterial cause. Severe dengue and severe malaria are two examples of non-bacterial infections that can give rise to hyper-inflammatory sepsis-like conditions [69], and both of them bind to cell surface HS. For dengue, among other viruses, HS-derivatized DNA origami shells have been designed capable of encapsulating and sequestering the virus [70]. In the case of malaria, both the BAEBL and circumsporozoite proteins of *P. falciparum* bind to HS on the surface of host cells [71,72]. A low-anticoagulant LMWH, sevuparin, has been shown to inhibit rosetting, block merozoite invasion and to de-sequester infected erythrocytes in humans with *P. falciparum* malaria [73,74].

Of the many viruses that bind cell surface heparan sulphate, the most intensively studied at present are from the SARS-CoV family, particularly SARS-CoV-2, the infectious agent of the current COVID-19 pandemic. The most serious stage of this condition involves a hyperinflammatory state sufficiently closely resembling bacterial sepsis to serve as an example of viral sepsis [75]. The viral invasion of host cells involves HS as an initial attachment factor, before the binding and internalisation consequent on interaction with the angiotensin converting enzyme (ACE-2) viral receptor. The SARS-CoV-2 spike protein interaction with cell surface HS has been investigated in detail, as summarised in a recent review [76]. It was rapidly determined that heparin was capable of inhibiting SARS-CoV-2 infection of cells by competing with HS for binding to the spike protein [10]. There is no evidence for selectivity for any HS structural motif [77], and indeed numerous different sulphated polymers have been shown to compete with HS binding to SARS-CoV-2 spike protein including marine sulphated glycans [78,79], pentosan polysulphate (a semi-synthetic sulphated xylan) [51] and others [80].

Direct delivery of heparin to the airway using a nebulizer has been shown to improve lung function in patients suffering from chronic obstructive pulmonary disorder (COPD) [81] and is therefore an attractive possibility for treatment or prophylaxis of COVID-19 by competing with cell surface HS [82]. LMWH administered by nebulization was found effective in protecting human nasal cells from infection with SARS-CoV-2, suggesting its possible prophylactic use [83]. Clinical trials of nebulized heparin in COVID-19 treatment are currently under way in several countries [11,12,13,84].

### 3.2. ‘Cytokine Storm’ in Sepsis

A central event in the development of sepsis is the ‘cytokine storm’ (CS) characterized by elevated circulating cytokine levels, acute systemic inflammatory symptoms, and leading to severe secondary organ dysfunction; sepsis is the most common cause of CS [85,86]. Both pro- and anti-inflammatory cytokines are involved, leading to a complex situation from which both hyperinflammation on the one hand, and immune suppression on the other, can result at different times in the course of the disease [87].

HS and heparin can interact with many cytokines (chemokines, interleukins and growth factors), and the anti-inflammatory activity of heparin has been ascribed at least in part, to the neutralisation of cytokine activity [88]. HS attached to proteoglycans in the extracellular matrix can modulate the diffusion of cytokines, chemokines and growth factors. Exogenous heparin can act by disrupting the leukocyte-directing cytokine gradients so created, and also may either enhance (as for IL-12 [89]) or inhibit (as for IL-6 [90]) functional interaction between cytokine and receptor [91].

Although studies of CS often concentrate on a few pro-inflammatory cytokines (for example IL-1β, IL-6, TNF-α), transcriptomic analyses have identified many more, and also followed the changing profile of cytokine expression over time during sepsis/endotoxemia [92].

It is therefore not easy to predict the consequences of treatment with heparin or one of its mimetics on the numerous cytokines released in CS, and the literature on this type of intervention is sparse. However, in the case of severe COVID-19 patients, a retrospective study has found significantly reduced IL-6 levels in LMWH treated patients, prompting the suggestion that this treatment may partially attenuate COVID-19 induced CS [93].

### 3.3. Neutrophils and Related Leukocytes in the Pathogenesis of Sepsis

Part of the pathogenesis of sepsis, and in many respects the pre-sepsis inflammatory response, centres around the innate immune response. A central element of innate immunity is the response to stimuli of neutrophils which have a central role in early host defence following infection [94]. Neutrophils, being the most abundant granulocytes [95] in the blood stream can respond in three principal ways following stimuli—phagocytosis of the foreign agent, degranulation which releases antimicrobial proteins and programmed cell death to form neutrophil extracellular traps (NETs) via a process known as NETosis (see below). These NETs ‘trap’ and destroy microbes [96]. The triggering of NETosis is considered highly likely in sepsis patients given the link between coagulation and inflammation (see below) and the occurrence of DIC in sepsis [97]. The term immuno-thrombosis may be applied in sepsis to indicate an inflammatory response coupled with altered/reduced coagulation factors; this aspect of sepsis has been reviewed recently [98,99,100]. The combination of uncontrolled inflammatory and coagulation responses can lead to organ damage and failure.

Neutrophils are therefore a logical target for the treatment and perhaps prevention of sepsis by attenuation of their release and activity. The results of the HETRASE trial [101] suggested the reduction in leukocyte activity may have contributed to the lack of mortality benefit observed for the unfractionated heparin treatment. There should therefore be some caution in that reducing neutrophils/leukocyte activity, the underlying cause of sepsis may no longer be targeted by the immune response. However, despite this observation with heparin, there is much interest in non-anticoagulant versions of heparin which retain effectiveness in targeting neutrophil response. The attenuation of neutrophils by heparin, and related mimetics, has been the subject of much interest in inflammatory pulmonary conditions [102,103] and treatment for COVID is a topical example [14]. Heparin has shown beneficial effects in reducing degranulation, disrupting NETs and cell–cell interactions; these roles have a potential therapeutic application in sepsis as described briefly below.

#### 3.3.1. Adhesion, Aggregation and Platelet Interaction

Heparin has been shown to be able to influence neutrophil aggregation [104,105,106,107] and interactions/aggregation with platelets [104,108]. Disruption of aggregation appears to take place via heparin interference with platelet P-selectin [109] and leukocyte L-selectin [110] and would reduce inflammatory activity through reducing platelet mediated leukocyte inflammatory responses [111]. These interactions are also involved in the activation of neutrophils [112] and the release of NETs [113,114]. Furthermore, in inflammation models in vivo heparin attenuated leukocyte adhesion, recruitment and invasion (see below for a discussion of the role of heparanase in invasion [110,115]). Again, this is likely to be the result of reducing L-selectin binding to endothelial cell heparan sulphate during leukocyte rolling and adhesion [116] and disruption of interactions with P-selectin on endothelial cells [117,118]. Interestingly, heparin has limited effect on endothelial cell E-selectin [110] expressed following stimulation.

Some structural determinants of the activity of heparin to prevent selectin mediated cell interactions have been determined. The 6-*O*-sulphate on glucosamine in heparin appears to have a role, along with a generally high degree of sulphation [119]. Modified non-anticoagulant heparin derivatives can also retain effectiveness [120,121] indicating that there may be further structural features that enhance the disruption of platelet–leukocyte interactions; for example parnaparin, a LMWH, is more effective than unfractionated heparin [122].

#### 3.3.2. Neutrophil Degranulation and Elastase Release

Prevention of adhesion and cell–cell interaction act as limiters on the first steps in neutrophil recruitment [123,124], but heparin can also influence the later activation of neutrophils. Heparin binds to the surface of neutrophils which limits degranulation [125], the production of superoxide anions and the activity of lysosomal enzymes [104] and the release of elastase [126]. Direct inhibition of elastase was also found to be effective with LMWH and *O*-desulphated heparin in experimental models [105,127] with a decasaccharide being optimal; however, this monodisperse fraction did not prevent cell adhesion, unlike heparin [125]. Plant-derived heparin mimetics such as fucoidan and xyloglucan can also neutralise elastase activity and have the added benefit of limited anticoagulant activity [128]. One potentially crucial aspect in attenuating elastase is the prevention of elastase inactivation of TFPI, an important inhibitor of coagulation [129] and limiting the activation of coagulation zymogens [130]. Lastly, the low anticoagulant LMWH sevuparin has been determined to neutralise cationic proteins, such as cathepsin G, elastase and the aptly named heparin binding protein (HBP), all of which may be released from neutrophils and increase vascular permeability [131]. Circulating levels of HBP correlate well as a predictive marker for sepsis [132].

#### 3.3.3. Neutrophil Extra Cellular Traps (NETs)

Neutrophils have been shown to generate NETs as an immune response at the sites of bacterial and viral infections [133,134,135]. These traps are composed of decondensed chromatin (DNA and histones) released from the nucleus and granular components (such as elastase, cathepsin G, Myeloperoxidase) which form fibrous networks to trap and destroy pathogens [135]. In a number of diseases, such as systemic lupus erythematosus [136], preeclampsia [137] and small vessel vasculitis [138], a pathological nature of NETs has been proposed. Such an uncontrolled inflammatory response occurs in sepsis and likely gives rise to excessive NET formation [139]. Markers of NETs such as cell-free DNA and histones have been shown to be increased in septic patients [140]. Furthermore, levels of histones correlate with sepsis severity [141], along with cell-free DNA [142,143].

The formation of NETs can be disrupted by heparin [144] which sequesters histones from NETs, exposing the DNA to degradation by DNAses in circulation [145] resulting in NET breakdown. A prothrombotic and anti-fibrinolytic effect has been shown by NETs [146,147] which can be disrupted by heparin, thereby reducing localised thrombosis [148,149]. These inhibitory effects are broadly retained by modified non-anticoagulant heparins [150,151,152].

The level of histones, unbound in NETs, in septic patients can be 200-fold or more compared to healthy volunteers [141]. Histones have multiple negative effects, activating platelets [144], stimulating tissue factor expression in endothelial cells and monocytes [153,154] and will bind to capillary glycocalyx leading to cell and organ damage [155]. Histones bind to cell surface heparan sulphate, and the interaction can be prevented by treatment with heparinase-III or addition of exogenous heparin or heparan sulphate, as shown in an experimental acute lung injury model [156]. Heparin and a non-anticoagulant heparin in a septic mouse model demonstrated a protective effect by blocking circulating histones [152]. In a histone-infused rat model where heparin has been shown to reduce histone induced cytotoxicity and inflammation, Zhu et al. [157] claimed that the attenuation effect of heparin was mediated through inhibition of calcium influx. Heparin or modified heparins limit cellular response to histones through acting as a heparan sulphate mimetic [53]. Sequestration of histones by heparin appears to be a charge-based interaction [158,159].

Preventing histone stimulation inhibits complement activation [150] and the release of cytokines such as IL6 from endothelial cells [160]. Activated platelets can stimulate neutrophils to undergo NETosis [113,161,162,163,164]. Given that heparin and non-anticoagulant derivatives and mimetics can inhibit the activation of platelets and binding of activated platelets to neutrophils, these agents could be viewed as a potential broad inhibitors of NETosis and NETs, and therefore warrant further investigation for their benefit in the treatment of sepsis.

### 3.4. Heparanase and Sepsis

The endoglucuronidase heparanase-1 (HPase) has been the subject of much recent interest. Though the literature has in the past been principally concerned with the identification of HPase as a target in the development of anti-cancer therapies (for example see [165]), several recent reviews have emphasised its role in the degradation of endothelial glycocalyx, particularly in sepsis [166,167,168,169,170]. HPase 1 is the only mammalian enzyme that breaks down heparin/HS; Heparanase-2 is a protein homologous to HPase, but in spite of its name it acts as an inhibitor of HPase and therefore has a protective effect on the glycocalyx by preventing the shedding of HS fragments [171].

HPase cleaves the heparin/HS chain at the reducing side of GlcA between two N-sulphated, preferably also 6-*O*-sulphated glucosamine residues [165]. This yields moderately large oligosaccharides consisting of about 10–20 monosaccharides [172,173]. In mast cells, heparanase cleaves heparin from its macromolecular form attached to the PG serglycin [174,175]. These HPase products are purified and used in medicine; they are relatively (though not completely) resistant to further HPase degradation and can bind and competitively inhibit the enzyme [174].

Though HPase is not abundant in most tissues under normal circumstances, its expression and activation are increased in tumours and in inflamed tissues; see ref [176] (and papers cited therein). Platelets are a cellular source of heparanase, with expression and activity of HPase increased in sepsis [172,173,174,175,176,177]. Endothelial heparanase degrades HS from the endothelial glycocalyx, resulting in local vascular injury and releasing highly sulphated, heparin-like HS oligosaccharides into the plasma [166]. In the course of this process, HPase also releases HS-bound growth factors, cytokines, and chemokines that are sequestered by heparan sulphate in the glycocalyx and in the extracellular matrix [178]. In addition, HS oligosaccharides generated by HPase can activate TLR-4 and thus cause the release of pro-inflammatory cytokines [179].

Endothelial dysfunction and impaired microcirculation are correlated with the severity and duration of sepsis [170] and have been shown to play a part in inflammatory injury to several different organs and tissues in pre-clinical studies as in the following examples.

Intravital microscopy was used to study the pulmonary glycocalyx in endotoxemic mice, identifying degradation via TNF-α dependent mechanisms including the activation of HPase. Inhibition of HPase with heparin (administered 3h after endotoxin challenge) prevented glycocalyx loss and neutrophil adhesion, and attenuated sepsis-induced acute lung injury (ALI) and mortality in mice [180]. In epithelial cell models of lipopolysaccharide (LPS)-induced acute respiratory distress syndrome (ARDS), expression of HPase was increased; *N*-acetylated heparin reduced LPS-induced HS degradation and tight junction damage [181]. HPase also contributes to septic acute kidney injury, and competitive inhibitors of HPase (heparin and a non-anticoagulant heparin) reduce the loss of glomerular filtration [182].

Intestinal injury in a mouse model of sepsis was reduced after inhibition of heparanase by unfractionated heparin [183]. A non-anticoagulant heparin was also found to attenuate intestinal injury, inhibit neutrophil infiltration and to suppress the production of inflammatory cytokines in a similar model [184].

In gram-negative bacterial sepsis, circulating high mobility group box 1 protein (HMGB1) binds extracellular LPS and mediates the cytosolic delivery of LPS into the cytoplasm of cells, leading to the activation of caspase-11. Through its ability to disrupt the HMGB1-LPS interaction, heparin can prevent caspase-11-dependent immune responses and lethality in endotoxemic mice, independently of its anticoagulant properties [185].

A synthetic ^13^C-labeled, highly sulphated HS nonasaccharide was used to determine rapid clearance of HS fragments from circulation, in a murine sepsis model. This nonasaccharide selectively targeted and penetrated the hippocampal blood–brain barrier following sepsis, rather than the cortex and other non-neuronal tissues [186]. Levels of circulating highly sulphated HS fragments have been found to be correlated with cognitive impairment in sepsis patients [187].

#### 3.4.1. Heparin Mimetics as Heparanase Inhibitors

Heparin mimetics are highly sulphated, structurally distinct analogues of glycosaminoglycans [188], that may be synthetic, naturally occurring or frequently semi-synthetic in origin. A recent review on the subject of HPase inhibitors [189] can be recommended as a good introduction to this field, describing several heparin derived or heparin mimetic HPase inhibitors: naturally occurring sulphated polysaccharides, semi-synthetic heparin mimetics based on bacterial HS-like polysaccharides, heparin modified by depolymerisation, supersulphation and desulphation, and ‘glycol-split’ heparins (in which the cis-diol in GlcA residues has been cleaved by periodate treatment, reducing anticoagulant activity). Several varieties of glycol-split heparin have reached clinical trials [189], though not for sepsis. One of these preparations, roneparstat, is an effective heparanase inhibitor and has recently been reviewed [190].

#### 3.4.2. Heparanase in Viral Sepsis

The severe COVID-19 associated endothelial dysfunction with cytokine release syndrome [82] is sufficiently similar to sepsis to be discussed here. Endothelial cells are a target of the SARS-CoV-2 virus, which induces both virus-mediated apoptosis and disruption of the glycocalyx. Heparin and its mimetics are under investigation in the development of therapeutic approaches to this syndrome [82]. HPase activity is elevated in COVID-19 patients, and is associated with disease severity, and prophylactic LMWH was associated with reduced HPase activity [191].

In the context of viral sepsis, roneparstat has been found to reduce SARS-CoV-2 related inflammatory cytokine release from human macrophages, and to decrease viral infectivity towards Vero-E6 cells, so demonstrating dual potential mechanisms of action in the treatment of COVID-19 [192]. Similarly, the heparin mimetic pixatimod has both HPase [193] and anti-viral infectivity [194] properties. HPase has also been identified as a contributor to the coagulopathy seen in COVID-19 patients [195].

## 4. Clinical Trials of Heparin in Sepsis

Clinical trials of heparin in sepsis have been empirical in their approach. In a 2017 review of experimental and clinical data, Li and Ma [196] found that evidence for the beneficial use of heparin in the treatment of sepsis was contradictory, possibly as a consequence of unsatisfactory meta-analysis of largely non-randomized trials. They were, however, on the whole optimistic about the prospects for heparin in reducing mortality in sepsis. A 2019 summary of systematic reviews [197] also found the overall impact of heparin unclear, suggesting that population heterogeneity among trial subjects might be part of the problem. More recently, a meta-analysis of ten randomized trials of LMWH in sepsis found a reduction in inflammatory reactions, lower incidence of multiple organ dysfunction (MODS) and 28-day mortality rate [46]. Another meta-analysis, this time of unfractionated heparin in sepsis, identified reduction in 28-day mortality and MODS [45]. In severe COVID-19 a therapeutic dose of heparin, as compared with a prophylactic dose, was not found to have any benefit to critically ill patients [35]; in another study LMWH at a therapeutic dose reduced major thromboembolism and death among inpatients with COVID-19 with very elevated D-dimer levels, but this effect was not seen in ICU patients [36].

These results from recent trials encourage cautious optimism. Because heparin is a potent anticoagulant the highest dose that can be contemplated for clinical trial use is the therapeutic dose, typically about 1 mg/kg twice daily. It is conceivable that this regime may not provide a sufficient concentration of heparin in circulation to take advantage of anti-inflammatory mechanisms that rely on competition with endothelial cell surface HS. HS is abundant in the glycocalyx so its local concentration there is high. An exogenous HS mimetic such as heparin therefore needs to be present in excess to be maximally effective. Heparin derivatives and mimetics with reduced anticoagulant activities may be safe at much higher concentrations than heparin itself, as indicated in trials of heparin derivatives for conditions other than sepsis. For the modified heparin roneparstat, 300–400 mg/kg has been recommended on the basis of a Phase 1 trial [198]; 18 mg/kg/day was the dose used in a trial of the modified LMWH sevuparin [199] and 100 mg/kg of pixatimod was tolerated well [200]. The introduction of heparin mimetic compounds as anti-inflammatory agents in sepsis treatment might therefore complicate the use of anticoagulant heparin to treat coagulopathy. It may even be the case that a high dose of mildly antithrombotic heparinoid (such as danaparoid) could be effective in both inflammatory and coagulation contexts, but this is pure speculation; we have no way of testing this until one or more heparin mimetics are approved for anti-inflammatory use.

There are also ways of using heparin without allowing it to enter the circulation. One of these is the technique described above for extracorporeal removal of pathogens from the blood [65], in which the therapeutic heparin is immobilised on a column; another is the use of nebulised heparin to allow direct access to the lung epithelium and associated pathogens without entering the bloodstream [14].

## 5. Concluding Remarks

The preceding paragraphs have outlined some of the mechanisms that might give rise to antiseptic and anti-inflammatory activities of heparin. We still do not understand the proportional contributions of these mechanisms to the therapeutic benefits of heparin treatment in sepsis and related conditions. However, the combination of novel methods of administration with developments of anti-inflammatory heparin mimetics allow optimism that safe and effective heparin-based treatments for sepsis will become available in the future.

## Data Availability

No new data were created or analyzed in this study. Data sharing is not applicable to this article.
